# Development of the invasive candidiasis discharge [I Can discharge] model: a mixed methods analysis

**DOI:** 10.1007/s10096-022-04473-w

**Published:** 2022-08-25

**Authors:** Jinhee Jo, Truc T. Tran, Nicholas D. Beyda, Debora Simmons, Joshua A. Hendrickson, Masaad Saeed Almutairi, Faris S. Alnezary, Anne J. Gonzales-Luna, Edward J. Septimus, Kevin W. Garey

**Affiliations:** 1 Department of Pharmacy Practice and Translational Research, University of Houston College of Pharmacy, 4349 Martin Luther King Blvd, Health 2, Houston, TX 77204 USA; 2 University of Texas Health Science Center, Houston, TX USA; 3 University of Texas MD Anderson Cancer Center, Houston, TX USA; 4 Department of Pharmacy Practice, College of Pharmacy, Qassim University, Qassim, Saudi Arabia; 5 Department of Clinical and Hospital Pharmacy, College of Pharmacy, Taibah University, Medinah, Saudi Arabia; 6 Department of Population Medicine Harvard Medical School, Boston, MA USA; 7 Texas A&M College of Medicine, Houston, TX USA; 8 School of Biomedical Informatics, The University of Texas Health Science Center at Houston, Houston, USA

**Keywords:** Candida, Candidemia, Echinocandin, Rezafungin, Mixed methods study design, Transitions of care

## Abstract

Patients with invasive candidiasis (IC) have complex medical and infectious disease problems that often require continued care after discharge. This study aimed to assess echinocandin use at hospital discharge and develop a transition of care (TOC) model to facilitate discharge for patients with IC. This was a mixed method study design that used epidemiologic assessment to better understand echinocandin use at hospital discharge TOC. Using grounded theory methodology focused on patients given echinocandins during their last day of hospitalization, a TOC model for patients with IC, the invasive candidiasis [I Can] discharge model was developed to better understand discharge barriers. A total of 33% (1405/4211) echinocandin courses were continued until the last day of hospitalization. Of 536 patients chosen for in-depth review, 220 (41%) were discharged home, 109 (20%) were transferred, and 207 (39%) died prior to discharge. Almost half (46%, 151/329) of patients discharged alive received outpatient echinocandin therapy. Independent predictors for outpatient echinocandin use were osteomyelitis (OR, 4.1; 95% CI, 1.1–15.7; *p* = 0.04), other deep-seated infection (OR, 4.4; 95% CI, 1.7–12.0; *p* = 0.003), and non-home discharge location (OR, 3.9, 95% CI, 2.0–7.7; *p* < 0.001). The I Can discharge model was developed encompassing four distinct themes which was used to identify potential barriers to discharge. Significant echinocadin use occurs at hospital discharge TOC. The I Can discharge model may help clinical, policy, and research decision-making processes to facilitate smoother and earlier hospital discharges.

**Supplementary Information:**

The online version contains supplementary material available at 10.1007/s10096-022-04473-w.

## Background

The incidence of invasive candidiasis (IC), defined as bloodstream infections with *Candida* species, deep-seated infections involving intra-abdominal organs or the peritoneum, or osteomyelitis, is approximately 3–5 per 100,000 persons, with around 50% of those occurring in the intensive care unit (ICU) setting [1]. The attributable mortality rate in patients with candidemia is reported to be between 10 and 47% with an attributable cost of $40,000 per patient [2, 3]. Current guidelines from the Infectious Diseases Society of America (IDSA) recommend echinocandins as empiric and/or initial therapy for IC due to superior response rates, an increasing prevalence of azole-resistant *Candida* spp., a favorable safety profile, and few drug-drug interactions [4, 5]. However, currently available echinocandins are intravenous and require at least once daily dosing, presenting a challenge to use in the outpatient setting.

Our group previously conducted an antimicrobial stewardship audit of hospitalized patients receiving echinocandins [6]. We identified that echinocandins are frequently continued until the day of hospital discharge and/or continued as outpatient infusions after hospital discharge. However, a better understanding of barriers encountered during hospital discharge transitions of care (TOC) is required to implement this finding. Although TOC models have been developed and used for various populations, [7–10] there are no TOC models published for patients with IC.

The objective of this study was twofold to (1) better understand echinocandin hospital discharge TOC and (2) build a TOC model focused on discharge barriers in patients with IC. The study was conducted in two parts: Part one was an evaluation of echinocandin use at hospital discharge, and part two used a qualitative approach to build a TOC model for patients with IC. We anticipated that the increased TOC knowledge and the TOC model would help clinicians, and policymakers optimize hospital discharge procedures for patients who require continued use of echinocandins on an outpatient basis.

## Methods

### Evaluation of echinocandin use

#### Study population

This multicenter retrospective study included hospitalized patients ≥ 18 years of age from two large Houston, Texas, area health systems (22 hospitals in total) between 2017 and 2019. Patients were prescribed echinocandins at the discretion of the treating medical team. This study was approved by the University of Houston Committee for the protection of research subjects with a waiver of informed consent.

#### Antibiotic use data

Pharmacy data from patients receiving any echinocandin antifungal (anidulafungin, caspofungin, or micafungin) were obtained through the Epic electronic health record (EHR) (Epic System Co., Verona, WI). Hospital census data were collected and paired with antibiotic use to assess rates of echinocandin use over time.

#### Cohort study

Patients who received an echinocandin for ≥ 48 h, including a dose on the last day of hospitalization, were identified, and one third of the cohort was randomly selected for in-depth EHR review by infectious diseases experts. Relevant demographics and clinical and microbiologic data were collected, including echinocandin indication, culture source, *Candida* species (if applicable), organism susceptibility, length of echinocandin therapy, and discharge disposition. Inpatient and outpatient medical records were evaluated to determine antifungal use following discharge. For those discharged with an echinocandin, an antifungal stewardship assessment was conducted including review of susceptibility of the infecting organism, drug-drug interaction(s), and drug toxicities, to determine appropriateness of outpatient echinocandin use.

#### Analysis plan

Descriptive statistics were calculated for patients receiving echinocandin therapy as inpatients and outpatients. Univariate and multivariable analyses were used to identify independent predictors of outpatient echinocandin use following hospital discharge. Variables with a *p* value < 0.2 in univariate analysis were included in a multivariable analysis, in which those with a *p* value < 0.05 were considered significant. R software version 4.0.3 (R Foundation for Statistical Computing, Vienna, Austria), STATA software version 13.0 (StataCorp LLC., College Station, TX), and/or SAS Version 9.3 (SAS Institute, Cary CN) were used for analysis.

### Development and assessment of an invasive candidiasis TOC model

#### Invasive candidiasis (I Can) discharge model development

A patient-centered component and an experienced-provider component were used to develop the invasive candidiasis (I Can) discharge model. Full methods detailing steps taken within each approach are available in the Supplementary Appendix. Briefly, the patient-centered approach used the previously identified subgroup of patients who received an echinocandin for ≥ 48 h (including on the last day of hospitalization) and an expert clinician evaluation to identify barriers that may have prevented hospital discharge. These barriers were then validated and organized into themes. In the experienced-provider approach, an open-ended, electronic survey (Qualtrics, Seattle, WA) based on a patient case of an adult with invasive candidiasis was sent to healthcare providers across the USA. Responses were recorded anonymously and analyzed through axial coding to develop thematic codes as previously described [11]. The codes were compiled and translated into a preliminary model that was finalized following several validation steps. Themes identified through both the patient-centered and experienced-provider approaches were incorporated into one final model, termed the I Can discharge model, with barriers categorized into four thematic categories.

#### I Can discharge model need assessment

An assessment was conducted in the cohort of patients from part one of the study that received in-depth chart review to estimate the proportion of patients pending discharge solely for TOC-related reasons. The study team conducted retrospective, daily assessments to identify the presence of barriers to discharge categorized as “medical course considerations” on each of the 3 days prior to discharge (days -1 to -3) in reverse chronological order. The earliest day that barriers were resolved was recorded, and the date of discharge TOC process initiation was identified. Discharge TOC processes may have included placement of a discharge order, consultation of case management service, placement of home health order, statement that patient is stable for discharge recorded anywhere in the notes, or any other mention of discharge preparation in the notes. The earliest day in which no “medical course consideration” barriers to discharge were present and a hospital discharge TOC process had begun was determined. The proportion of patients with no barriers to discharge within each of the model’s four thematic categories was calculated for each day and aggregated to determine the potential date a patient may have been discharged if no echinocandin-related TOC barriers were present. Baseline premises applied in the need assessment and interpretation included that TOC-related barriers were primarily due to the use of continued IV antimicrobial requiring arrangement of home health infusion services prevented earlier discharge.

## Results

### Evaluation of echinocandin use

A total of 4211 echinocandin courses were evaluated with a total echinocandin days of therapy (DOT) of 22,888 days. The median length of hospital stay was 18 days (IQR, 9–32 days). The median time from hospital admission to echinocandin initiation was 5 days (IQR, 1–12 days), and the median length of therapy was 3 days (IQR, 1–6 days). Overall, 1405 (33%) echinocandin courses were continued until the last day of hospitalization. Approximately one third (38%, *n* = 536) of these patients were randomly selected for inclusion in a convenience sample subgroup to undergo in-depth chart review.

These 536 patients were aged 58 ± 16 years (57% male, 67% white race). All patients received only micafungin, and the most frequent indications for use were intra-abdominal cultures positive for *Candida* species (23%, *n* = 124), suspected IC without positive cultures (14.9%, *n* = 80), or candidemia (8.6%, *n* = 46). The majority of patients (93%, *n* = 498) received 100 mg of micafungin administered once daily (standard dosing), while 37 (7%) patients received 150 mg daily and one (0.2%) patient received 200 mg daily. Thirty-three (6%) patients received more than one course of micafungin during hospitalization; of these, 7 (21%) were given standard dosing, while 26 (79%) were given 150 mg once daily (*n* = 21) or doses higher than 150 mg (*n* = 5). Micafungin inpatient DOT averaged 9 ± 9 days (median, 6 days; IQR, 3–11 days). Most patients (61%, *n* = 328) received ≤ 7 inpatient DOT, while 23% (*n* = 124) received 7–14 days and 16% (*n* = 84) received ≥ 14 days.

**Fig. 1:**
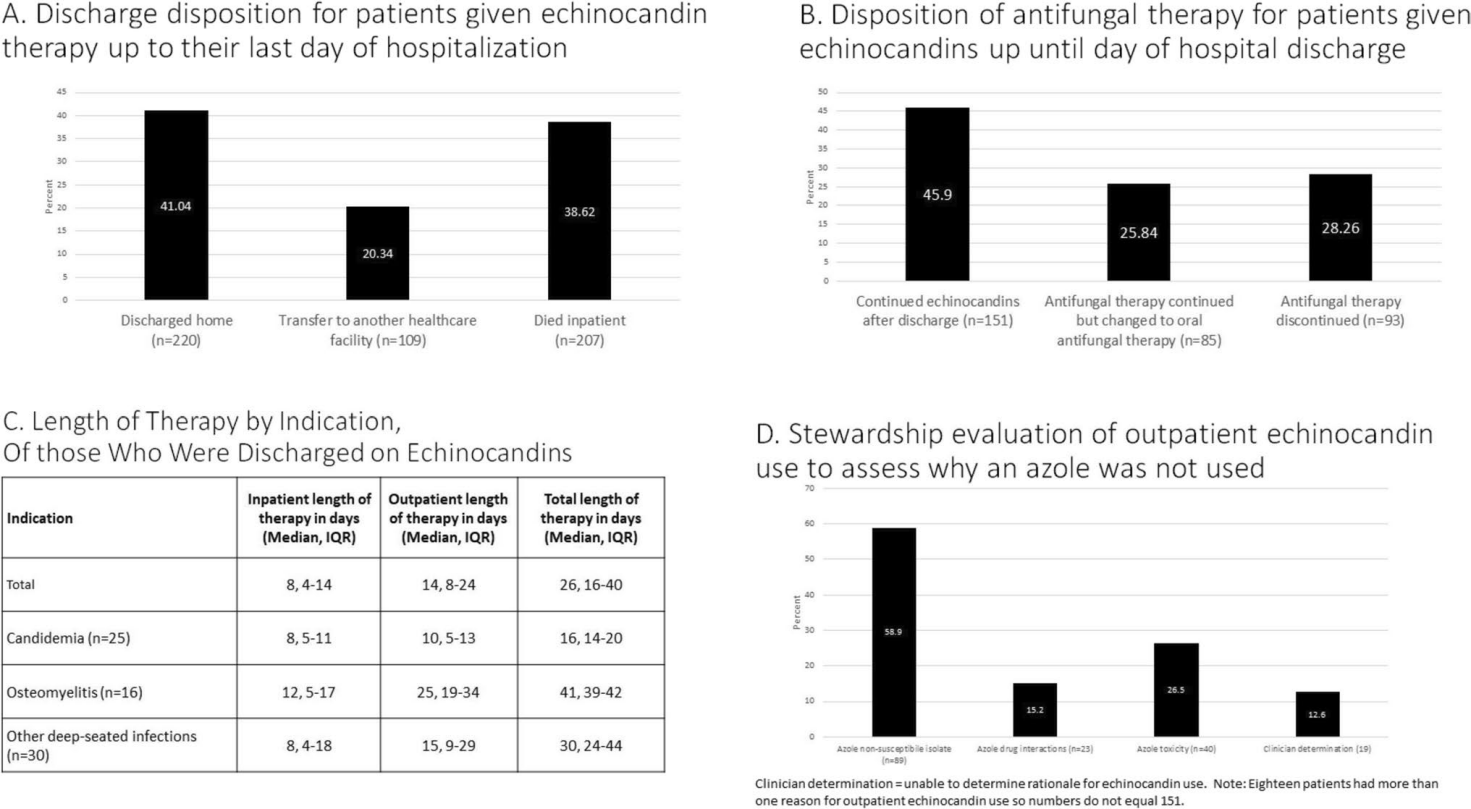
Summary of echinocandin evaluation findings. **A** Discharge disposition for patients given echinocandin therapy up until their last day of hospitalization. **B** Disposition of antifungal therapy for patients given echinocandin therapy up until the day of hospital discharge. **C** Length of therapy by indication of those discharged on echinocandin therapy. **D** Stewardship evaluation of outpatient echinocandin use to assess azole inappropriateness

Overall, 329 (63%) patients were discharged alive and were most commonly discharged home (67%, *n* = 220) (Fig. 1A). Almost half of these patients (46%, *n* = 151) continued echinocandin therapy on an outpatient basis, and a median outpatient DOT of 14 days (IQR, 8–24 days) was calculated for the 133 patients with information available (Table 1, Fig. 1B and 1C). The stewardship audit of these patients identified the most common reasons therapy was not changed to an oral antifungal as isolation of an azole-resistant *Candida* species (59%, *n* = 89), potential toxicity with an azole (27%, *n* = 40), or potential drug interaction(s) with an azole (15%, *n* = 23) (Fig. 1D).

**Table 1:** Univariate and multivariate results on predictors of outpatient use of echinocandins

		Discharged on an echinocandin					
		Univariate analysis			Multivariable analysis		
Variable	*N*	No (*n* = 178)	Yes (*n* = 151)	*P* value	OR	95% CI	*P* value
Age, years		54 ± 17	59 ± 15	0.0039			
Sex, female	136	64.0%	36.0%	0.003			
Race, White	236	68.0%	76.2%	0.101			
ICU anytime during admission	164	48.9%	51.0%	0.702			
Echinocandin initiation in ICU	127	37.6%	39.7%	0.615			
Azole administered concomitantly during hospitalization	28	10.7%	6.0%	0.127			
Culture positive for *Candida* spp.	179	43.8%	66.9%	< 0.0001			
*C. albicans*	69	18.0%	24.5%	0.147			
Non-*albicans Candida* spp.	130	31.5%	49.0%	0.0012			
Mixed (*C. albicans* + *C. glabrata*)	20	5.6%	6.6%	0.012			
Indication for echinocandin therapy							
Candidemia	46	10.1%	18.5%	0.028			
Intra-abdominal	124	36.5%	39.0%	0.634			
Esophageal candidiasis	6	66.7%	33.3%	0.533			
SSTI	20	40.0%	60.0%	0.192			
Osteomyelitis	21	1.7%	11.9%	0.0002	4.07	1.06–15.66	0.041
Respiratory	18	5.6%	5.3%	0.900			
Lung transplant prophylaxis	14	100.0%	0.0%	0.000			
Suspected IC	80	33.2%	13.9%	< 0.0001			
Other deep-seated infection	49	8.99%	21.9%	0.001	4.44	1.65–11.96	0.003
Inpatient echinocandin DOT				0.0002			
≤ 7 days	194	68.5%	47.7%				
8 to 14 days	81	21.4%	28.5%				
≥ 14 days	54	10.1%	23.8%				
Transfer to another healthcare facility	109	21.4%	47.0%	< 0.0001	3.89	1.95–7.74	0.000

Abbv: *ICU*, intensive care unit; *spp*, species; *SSTI*, skin and soft tissue infection; *IC*, invasive candidiasis; *DOT*, days of therapy

**Fig. 2:**
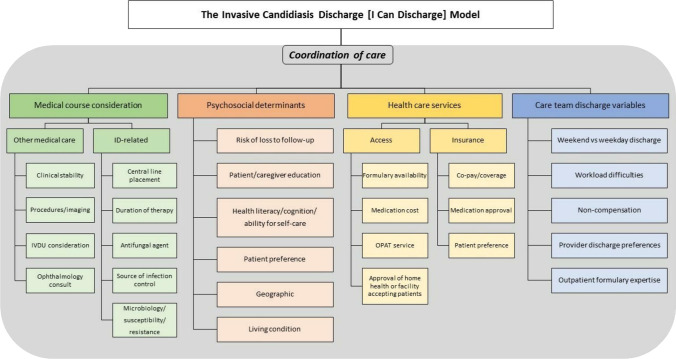
The invasive candidiasis (I Can) discharge model

A univariate analysis was conducted and followed by multivariable modeling to identify patient characteristics associated with a higher likelihood of continuing on echinocandin therapy after hospital discharge (Table 1). In univariate analysis, age, female sex, certain indications for use (candidemia, osteomyelitis, lung transplant prophylaxis, other deep-seated infections), positive microbiology results for any *Candida* species or non-albicans species, inpatient echinocandin DOT, and non-home discharge location were associated with a higher likelihood of continuing echinocandins after hospital discharge. The multivariable model identified osteomyelitis (OR, 4.1; 95% CI, 1.1–15.7; *p* = 0.04), other deep-seated infection (OR, 4.4; 95% CI, 1.7–12.0; *p* = 0.003), and a non-home discharge location (OR, 3.9, 95% CI, 2.0–7.7; *p* < 0.001) as significant independent predictors for echinocandin outpatient use.

### Development and application of an IC TOC model

The invasive candidiasis discharge model was composed of four distinct themes: medical course considerations, psychosocial determinants of health, healthcare services, and care team discharge variables (Fig. 2). An over-arching value of the I Can discharge model was effective coordination of care between and within the four distinct themes.

**Fig. 3:**
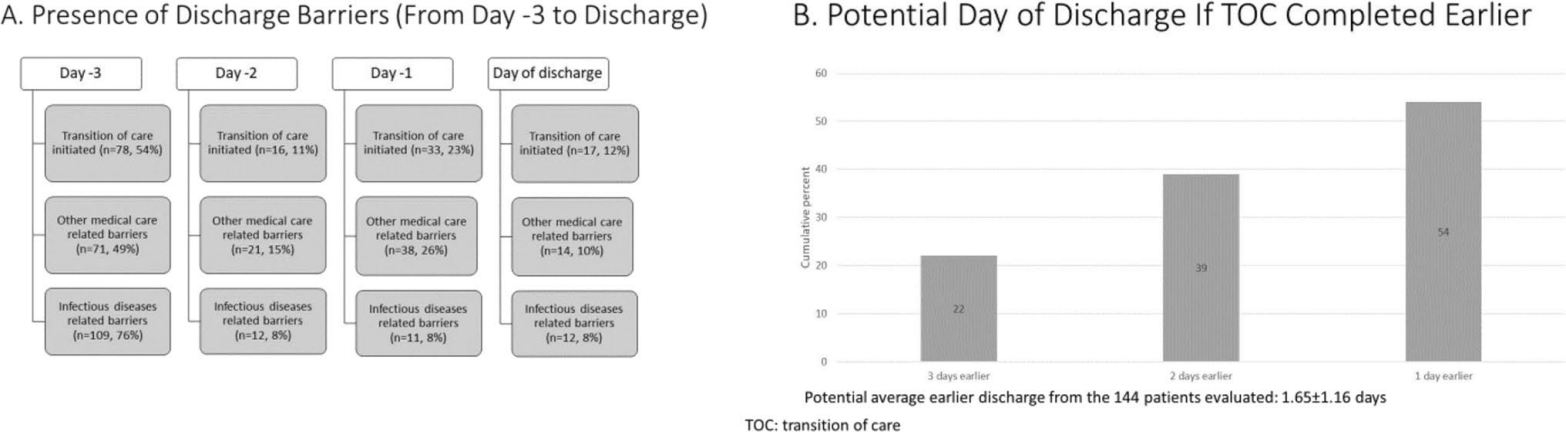
Need assessment of discharge barriers present in the 3 days prior to discharge. **A** Each day represents whether initiation of transition of care (top row) or presence of medical course consideration barriers (other medical care-related (second row) and infection disease-related (third row)) were present. **B** Using the I Can discharge model, summary estimates on excess length of hospital stay due to IV antimicrobial

A total of 144 patient cases were analyzed in the need assessment to estimate the excess length of hospital stay based on continued need for outpatient echinocandin therapy (Fig. 3). The TOC process was initiated prior to the day of discharge in 127 (88%) patients, and more than half of patients (54%, *n* = 78) had TOC processes initiated on day -3 from discharge (Fig. 3A). Although the majority of patients had medical course consideration-related barriers present on day -3, 98 (68%) patients had resolution of infectious diseases-related barriers, and 33 (39%) patients had resolution of medical care-related barriers prior to their actual date of discharge. A minority of patients (22%) had a potential discharge date on day -3 from actual discharge, based on the presence of TOC process initiation and absence of medical course consideration-related barriers (Fig. 3B). This increased to 39% of patients with a potential discharge day 2 days earlier and 54% of patients with a potential day 1 day earlier. Taken together, the average excess length of stay due to echinocandin-related TOC barriers was 1.7 ± 1.2 days.

## Discussion

TOC discharge barriers negatively impact clinical outcomes and extend hospital length of stays in patients with a variety of infectious diseases [12–14]. Using an epidemiologic, multihospital analysis of over 4,000 echinocandin courses, this study demonstrated that one third of all echinocandin courses in hospitalized patients were continued until the last day of hospitalization, and approximately one half of patients discharged from the hospital continued echinocandin therapy. Using a qualitative mixed methods study design and a national group of experts, this study created the first TOC discharge model for patients with (the I Can discharge model) to help identify four themes of potential discharge barriers for patients requiring outpatient echinocandin use. A need assessment demonstrated more than half of patients with IC may have been discharged 1 day earlier, indicating a large potential niche of patients of whom may benefit from implementation of the I Can discharge model.

The study design identified barriers to discharge through two separate approaches from differing perspectives, later integrated to form the I Can discharge model. These two perspectives yielded barriers of similar themes, indicating a high level of internal validity for our final model. In addition, the model was developed based on data from local hospital systems in Houston, Texas, and from a national cohort of healthcare professionals practicing at various centers throughout the USA. Incorporating both local and national perspectives increases the external validity and transferability of the I Can discharge model. Finally, through conducting a need assessment involving real patients, we demonstrated that hospital length of stay could be potentially shortened with improvements in the TOC process. Healthcare organizations, policy makers, and researchers can use this model to help identify discharge barriers for patients with IC, allowing optimization of discharge planning and improvements in TOC for this highly complex patient population.

Many of the TOC barriers present in patients with IC are related to the continued need for IV echinocandin therapy. Importantly, a number of newer generation echinocandins are in development, including rezafungin, a cyclic hexapeptide derived from anidulafungin characterized by increased in vivo stability and a longer half-life allowing for higher doses and once weekly dosing [6]. The recently published Phase II STRIVE trial demonstrated that rezafungin has comparable safety and efficacy to caspofungin in the treatment of candidemia and/or IC, providing an exciting potential addition to current echinocandin antifungals [15]. Future studies are needed to assess potentially reduced costs and length of stays associated with this new agent.

TOC models from other disease states helped inform development of the I Can discharge model and provide ideas for future areas of development. Hospital discharge barriers have been shown to impact patient care, increase costs, and lead to poor outcomes [12–14]. There are a number of TOC models that have demonstrated changes in these outcomes after implementation. For example, application of a TOC model for hospitalized, homeless patients were able to reduce hospital re-admission rates [8]. Related to our study, the use of a TOC OPAT model helped develop targeted solutions facilitating transitions in both the inpatient and outpatient stages for patients requiring antibiotic therapy [16]. Prior studies have also recognized several themes as high priorities for TOC planning, including two of the major themes identified during the development of the I Can discharge model—effective communication between the team coordinating the discharge process and barriers to antifungal decision-making [17, 18]. Finally, a qualitative study of pediatric patients’ family’s perspectives identified several novel areas that should be included in TOC planning [19]. Future iterations of the I Can discharge model should include a patient or caregiver perspective as well.

This study has certain limitations. The echinocandin evaluation was based on data collected from two large and distinct healthcare systems in the greater Houston area. Validation of these findings will be required in a nationwide cohort. As highlighted in the I Can discharge model and elsewhere, coordination of care must exist within inpatient specialties as well as between outpatient services in the discharge process. The I Can discharge model aims to facilitate this coordination by identifying TOC-related barriers and allowing for improved patient care surrounding TOCs.

In conclusion, this study used a mixed methods design to demonstrate significant TOC barriers for patients with IC receiving echinocandins during hospital discharge. The I Can discharge model was developed to facilitate healthcare services, inform policy makers, and serve as a tool for future studies in order to improve the continuation of care for this complex patient population. A need assessment was used to demonstrate the utility of the model in potentially shortening the length of stay. Given these results, the I Can discharge model may help facilitate clinical, policy, and research decision-making processes to facilitate smoother and earlier hospital discharges.

## Supplementary Information

Below is the link to the electronic supplementary material.

Supplementary file1 (DOCX 15 KB)

Supplementary file2 (JPEG 149 KB)

## Data Availability

Datasets generated during this study are available in the supplementary material for this manuscript.
