# Supplementary material for: Development of the invasive candidiasis discharge [I Can discharge] model: a mixed methods analysis
Source: Eur J Clin Microbiol Infect Dis. 2022 Aug 25;41(10):1207–13. doi: 10.1007/s10096-022-04473-w (PMC9489576; doi:10.1007/s10096-022-04473-w)
Supplement: Supplementary file 1 — Supplementary file1 (DOCX 15 KB) [file 10096_2022_4473_MOESM1_ESM.docx]

Supplementary appendix

Methods to Develop the Invasive Candidiasis Discharge Model:

*Patient-centered approach:* The research group used the previously identified subgroup of patients who received an echinocandin for ≥ 48 hours (including on the last day of hospitalization) to identify variables that may have prevented hospital discharge and assess whether the availability of an echinocandin administered once weekly would facilitate an earlier discharge. These barriers-to-discharge were compiled and used to create a data collection instrument in Excel (Microsoft Corp., Redmond, WA). Standardized definitions were created and distributed as a PDF Data Dictionary to research team members to assure consistent collection of data. Three iterative group exercises, each using five to ten patient chart reviews, were conducted to validate the elements, assess whether all data were available, and explore any missing variables. After each chart review, the instrument was adapted based on data availability and collaborative group feedback. Barriers were organized into themes based on group discussion. After these reviews, the instrument was converted into a final data collection tool in REDCap (Vanderbilt University, Nashville, TN).

*Experienced-provider approach*: A second, separate study methodology was used to validate the discharge-related barriers identified in the Patient-centered approach. An electronic survey (qualtrics, Seattle, WA) with seven, open-ended questions was created based on a fictional patient case of an adult with candidemia. Using the study team's professional connections, the survey was distributed via email to healthcare professionals across the United States in order to collect real-world data regarding the discharge processes for patients requiring outpatient echinocandin therapy. Responses were recorded anonymously and analyzed through axial coding to develop thematic codes as previously described.[12] The initial codes from three research team members (TTT, KWG, NDB) were compiled and translated into an initial model of discharge issues in patients with IC. A team of three infectious diseases experts (physician, pharmacist, nurse) from the study group then conducted independent, one-on-one, semi-structured, virtual interviews in which the responses to four, open-ended, model-related questions were used to further refine the model. Lastly, a panel of eight interdisciplinary infectious diseases experts was consulted to validate the model using a series of three semi-structured questions.
