# Supplementary figures and images for: Development of the invasive candidiasis discharge [I Can discharge] model: a mixed methods analysis
Source: Eur J Clin Microbiol Infect Dis. 2022 Aug 25;41(10):1207–13. doi: 10.1007/s10096-022-04473-w (PMC9489576; doi:10.1007/s10096-022-04473-w)

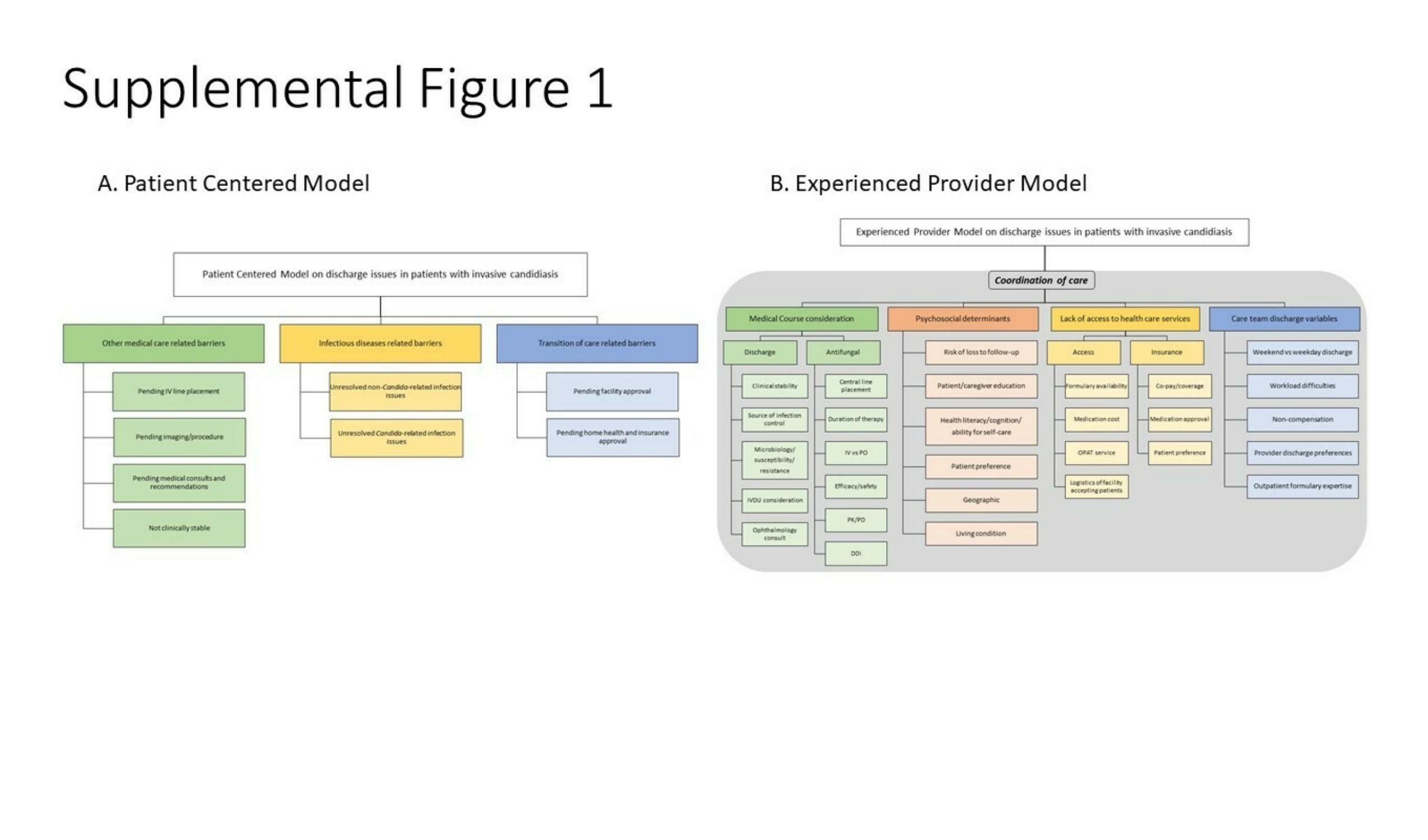

Supplement: Supplementary file 2 — Supplementary file2 (JPEG 149 KB) [file 10096_2022_4473_MOESM2_ESM.jpeg]
